# Multiple myeloma: routes to diagnosis, clinical characteristics and survival – findings from a UK population‐based study

**DOI:** 10.1111/bjh.14513

**Published:** 2017-02-01

**Authors:** Debra Howell, Alexandra Smith, Simon Appleton, Timothy Bagguley, Una Macleod, Gordon Cook, Russell Patmore, Eve Roman

**Affiliations:** ^1^ Epidemiology & Cancer Statistics GroupDepartment of Health SciencesUniversity of YorkYorkUK; ^2^ Centre for Health and Population SciencesUniversity of HullHullUK; ^3^ Department of HaematologySt James's University HospitalLeedsUK; ^4^ Queen's Centre for Oncology and HaematologyCastle Hill HospitalCottinghamUK

**Keywords:** multiple myeloma, route to diagnosis, clinical characteristics, survival, emergency presentation

## Abstract

Prompt cancer diagnosis may align UK survival with European averages. We examined the impact of route to diagnosis on survival for multiple myeloma patients diagnosed 2012–2013 using data from our population‐based patient cohort that links to national death notifications and collects details on treatment and response (*n* = 441). Emergency presentation was associated with advanced disease and poorer outcomes, and was the commonest route to diagnosis (28·1%) followed by General Practitioner urgent (19·0%) and two‐week wait (17·2%) referrals. CRAB (elevated Calcium, Renal failure, Anaemia, Bone lesions) distribution varied by route (*P* < 0·001), with patients with emergency presentations most likely to have ≥2 features and significantly worse survival (log‐rank test χ^2^ = 13·8, *P* = 0·0002).

Estimates suggest that each year in Britain, 6600–7500 premature cancer deaths could be prevented if survival matched European averages (Abdel‐Rahman *et al*, 2009). Around a quarter of these “avoidable” deaths are attributable to haematological malignancies, with multiple myeloma (MM) accounting for almost half. Earlier diagnosis has been identified as one factor likely to improve cancer outcomes (Thomson & Forman, 2009), and a series of interventions have been introduced into UK practice to promote this, including referral guidance to facilitate identification of cancer symptoms in primary care and a suspected cancer referral pathway (“two‐week wait”) (Department of Health, 2000; NICE, 2005). Whilst the former has expedited diagnosis of many cancers, average times for MM have increased (Neal *et al*, 2014).

An estimated 4,300 people are diagnosed with MM annually in the UK www.hmrn.org/statistics/incidence. Presenting symptoms are often vague and include musculoskeletal pain and tiredness; and patients are more likely than those with other cancers to have three or more General Practitioner (GP) visits before a secondary care referral is initiated (Lyratzopoulos *et al*, 2012; Howell *et al*, 2013). Delayed diagnosis of MM is associated with increased risk of complications (bone disease, anaemia and renal failure) and poorer survival (Kariyawasan *et al*, 2007; Friese *et al*, 2009). MM is also more likely than other cancers to be diagnosed after emergency presentation, a route considered a crude indicator of delay and associated with poorer outcomes (Elliss‐Brookes *et al*, 2012). This paper examines the impact of route to diagnosis on MM survival and clinical complications.

## Patients and methods

The study is set within the Haematological Malignancy Research Network (HMRN: www.hmrn.org), a UK population‐based cohort instigated in 2004 to generate ‘real world’ data for research and clinical purposes (www.hmrn.org/publications/papers). HMRN covers a population of around four million, with clinical care in the area adhering to national guidance (Smith *et al*, 2011). All diagnoses of haematological malignancy (>2200 annually) are made and coded using the latest World Health Organization (WHO) oncology classification (Swerdlow *et al*, 2016) by a single integrated haematopathology laboratory (the Haematological Malignancy Diagnostic Service: www.hmds.info). Following diagnosis, a core dataset is routinely abstracted from patients’ medical records. For MM this includes diagnostic imaging and blood tests, with complications and prognostic risk being assessed using components of the CRAB (elevated Calcium, Renal failure, Anaemia, Bone lesions) criteria (Rajkumar *et al*, 2014) and the International Staging System (ISS) (Greipp *et al*, 2005). With Section 251 support, all HMRN patients are tracked through clinical systems and linked to nationwide information on deaths.

For the present study, core data on myeloma patients diagnosed 1 July 2012 to 31 December 2013 were supplemented with information on routes to diagnosis. This included documentation of all referrals from the time the patient first presented to hospital with potential MM symptoms to diagnosis, defined as the date HMDS received the diagnostic sample. Data abstracted for each referral were: date/type of referral, clinical speciality referred from/to, and date of first appointment. Referral categories were based on the UK's National Cancer Intelligence Network (NCIN) study (Table SI).

Survival (from date of diagnosis) was calculated with standard time‐to‐event analyses, and the program strel (v1.2.7; http://www.lshtm.ac.uk/ncde/cancersurvival/tools.htm) was used to estimate relative survival. Age and sex‐specific background mortality rates were obtained from national life tables (Allemani *et al*, 2015), and all analyses were undertaken in Stata 14 (www.stata.com).

## Results

With a median diagnostic age of 74·2 years, 441 patients were diagnosed with MM during the study period. Overall, emergency presentation was the commonest route to diagnosis (28·1%, *n* = 124). Around two‐thirds of patients using the emergency route (*n* = 79) had been referred to hospital by a GP, either via Accident and Emergency (A&E) or a direct ward‐admission. Of the 45 without prior GP contact, 34 self‐referred to A&E or arrived via the actions of family members, nursing home staff, emergency services or the general public; and the remainder were referred from other hospital specialities (e.g. imaging, physiotherapy). After emergency, the next most frequent routes were GP urgent (*n* = 84, 19·0%), GP two‐week wait (*n* = 76, 17·2%), GP routine (*n* = 56, 12·7%) and hospital consultant‐to‐consultant (*n* = 41, 9·3%).

Overall, 60 patients (13·6%) had no referral route recorded, either because they were already being monitored by haematology (commonly for monoclonal gammopathy of undetermined significance, MGUS) (*n* = 39, 8·8%) or because no details were documented in their hospital records (*n* = 21, 4·8%). Demographic and clinical characteristics of the remaining 381 (86·4%) patients with a referral route are presented in Table 1. According to CRAB criteria, 114 (29·9%) of these patients were asymptomatic at diagnosis (score: zero), 139 (36·5%) had one CRAB feature and 125 (32·8%) had two or more. CRAB could not be calculated for 3 patients: one (aged >80 years) died from other causes, one (aged >80 years) opted for no tests/treatment and died at home, and another (aged >90 years) died from kidney disease. CRAB distributions varied markedly with referral route (*P* < 0·001), with patients presenting as an emergency being the most likely to have a CRAB score ≥2 (*n* = 70, 56·5%) and the least likely to have a score of zero (*n* = 11, 8·9%). Conversely, patients with routine referrals were least likely to have a CRAB ≥2 (*n* = 6, 10·7%) and most likely to be asymptomatic (*n* = 28, 50·0%). Findings for ISS showed similar variations, with ISS III (the most clinically advanced disease) being most commonly seen among emergency presentations (*n* = 48, 51·1%). ISS could not be calculated for 70 (18.4%) patients, largely because βeta_2_ microglobulin (β_2_M) was not measured.

**Table 1:** Referral route by demographic and clinical characteristics: HMRN myeloma diagnoses July 2012 to December 2013

	Referral route				
	Emergency	Two‐week wait	GP urgent	Routine	Consultant
	*N* (%)	*N* (%)	*N* (%)	*N* (%)	*N* (%)
Total	124 (100·0)	76 (100·0)	84 (100·0)	56 (100·0)	41 (100·0)
Sex					
Males	70 (56·5)	41 (53·9)	47 (56·0)	35 (62·5)	27 (65·9)
Females	54 (43·5)	35 (46·1)	37 (44·0)	21 (37·5)	14 (34·1)
Age at diagnosis (years)					
Median (IQR)	73·7 (65·4–79·5)	74·7 (64·1–80·9)	71·9 (64·2–80·2)	74·5 (68·7–81·7)	74·3 (67·3–78·7)
CRAB features					
0	11 (8·9)	27 (36·5)	35 (41·7)	28 (50·0)	13 (31·7)
1	42 (34·1)	32 (43·2)	29 (34·5)	22 (39·3)	14 (34·1)
≥2	70 (56·5)	15 (19·7)	20 (23·8)	6 (10·7)	14 (34·1)
Unknown	1	2	0	0	0
Hypercalcaemia					
Yes	22 (17·7)	5 (6·6)	1 (1·2)	3 (5·4)	5 (12·2)
No	102 (82·3)	71 (93·4)	83 (98·8)	53 (94·6)	36 (87·8)
Renal insufficiency					
Yes	43 (34·7)	3 (3·9)	9 (10·7)	2 (3·6)	9 (22·0)
No	81 (65·3)	73 (96·1)	75 (89·3)	54 (96·4)	31 (78·0)
Anaemia					
Yes	62 (50·0)	18 (23·7)	25 (29·8)	12 (21·4)	15 (36·6)
No	62 (50·0)	58 (76·3)	59 (70·2)	44 (78·6)	25 (63·4)
Bone disease					
Yes	85 (69·7)	41 (55·4)	35 (41·7)	19 (34·5)	21 (51·2)
No	37 (30·3)	33 (44·6)	49 (58·3)	36 (65·5)	20 (48·8)
Unknown	2	2	0	1	0
ISS					
I	9 (9·6)	28 (41·8)	21 (30·4)	19 (41·3)	9 (25·7)
II	37 (39·4)	21 (31·3)	26 (37·7)	20 (43·5)	9 (25·7)
III	48 (51·1)	18 (26·9)	22 (31·9)	7 (15·2)	17 (48·6)
Unknown	30	9	15	10	6
First referral to haematology	0 (0·0)	68 (89·5)	63 (75·0)	41 (73·2)	31 (75·6)
First line management					
Chemo/radiotherapy	96 (77·4)	45 (59·2)	49 (58·3)	24 (42·9)	24 (58·5)
Observation	10 (8·1)	26 (34·2)	26 (31·0)	28 (50·0)	15 (36·6)
Supportive/palliative	18 (14·5)	5 (6·6)	9 (10·7)	4 (7·1)	2 (4·9)

CRAB, elevated Calcium, Renal failure, Anaemia, Bone lesions; GP, General Practitioner; HMRN, Haematological Malignancy Research Network; IQR, interquartile range; ISS, International Staging System.

Information on haematology referrals and first‐line management is also presented in Table 1. Of two‐week wait referrals (to any clinical speciality), around 9 out of every 10 (68/76) were sent directly to haematology, as were around three‐quarters of those entering secondary care by all other routes except emergency. Patients with routine referrals tended to have fewer risk factors at presentation and around half were initially managed by observation. In contrast, 77·4% of emergency presentations received first‐line chemotherapy/radiotherapy and 14·5% were managed with supportive/palliative intent – both proportions being higher than any other diagnostic route.

**Figure 1:**
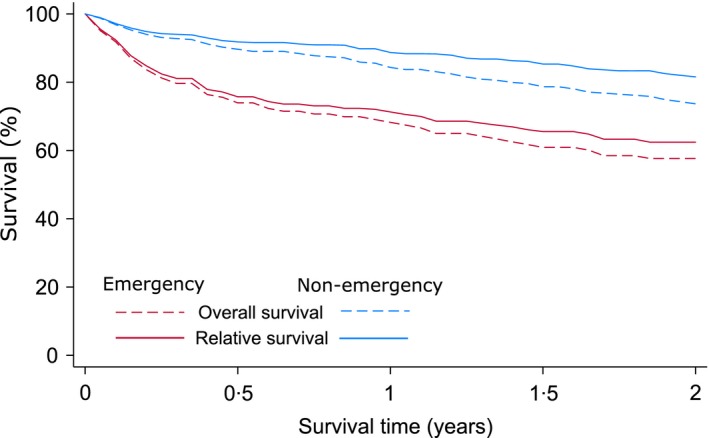
Two year overall and relative survival: Haematological Malignancy Research Network (HMRN) myeloma diagnoses July 2012 to December 2013.

Importantly, these differences translate into marked variations in outcome, with the overall and relative survival of patients with an emergency route being significantly worse than those presenting via other routes (Fig 1). As might be expected, the impact on survival is immediate; the overall survival (OS) and relative survival (RS) estimates of patients presenting as an emergency diverging markedly from that of other patients within 3 months of diagnosis: the 1 year RS estimates being 72·6% (95% Confidence Interval (CI) 62·9–80·2%) and 88·6% (95% CI 83·7–92·1%) for emergency and non‐emergency presentation respectively (log‐rank test χ^2^ = 13·8, *P* = 0·0002).

## Discussion

Multiple myeloma patients diagnosed via the emergency route were more likely to have clinically advanced disease, be managed with first‐line chemotherapy/radiotherapy or supportive/palliative intent and have the poorest survival. Conversely, around a third of patients presenting by other routes had asymptomatic disease and were initially managed with observation. The greatest impact on mortality was seen within the first 6 months of diagnosis, during which time around 1 in 4 patients who presented as an emergency died compared to less than 1 in 10 of those presenting via other routes. That these patients had more advanced/aggressive disease was evidenced by their significantly greater need for chemotherapy/radiotherapy as first‐line treatment. A small proportion of emergency presentations were, however solely observed, and while this may appear counterintuitive, these patients were mostly diagnosed incidentally during investigations for other serious co‐morbidities.

Determining when to monitor, actively investigate, or refer patients to hospital can prove challenging in primary care, particularly for cancers like MM that can present with vague symptoms and insidious onset. Nonetheless, GPs played a significant role in referrals, initiating almost two‐thirds of emergency presentations and a large proportion of non‐emergency referrals to haematology, suggesting prior identification of blood‐related abnormalities. Similarly, 90% of all GP two‐week wait referrals were direct to haematology, implying suspicion of haematological malignancy.

This is the first study to use secondary care data alongside demographic and clinical details to examine routes to diagnosis of MM. Major strengths include a large well‐defined population‐based catchment area, completeness of case ascertainment, detailed follow‐up and world‐class diagnostics. CRAB scores were largely complete, although ISS was missing for a fifth of patients because β_2_M was not always measured, particularly in older patients and those with a prior diagnosis of MGUS.

Detailed data of the type presented here are not available elsewhere for direct comparison. The UK NCIN study (Elliss‐Brookes *et al*, 2012), based on Hospital Episode Statistics, reported a higher proportion of emergency presentations and fewer two‐week waits; similar proportions of routine/urgent GP referrals combined; and poorer 1 year RS (51%, 95% CI 49·0–53·0). These differences may reflect the survival advantages of new treatments, or the way the date of diagnosis (e.g. report date) was assigned in our cohort compared to later dates often used in national data (e.g. first treatment date).

Our findings highlight the potential benefits of expediting MM diagnosis and minimising emergency presentations. This may be achieved through raising awareness about MM, both in health settings and among the general public. Furthermore, a better understanding of events in primary care would facilitate the development and testing of evidence‐based interventions to prevent emergency presentation.

## Ethics statement

HMRN has ethical approval (REC 04/01205/69) from Leeds West Research Ethics Committee, R&D approval from each Trust, and exemption from Section 251 (formally Section 60) of the Health & Social Care Act (2001) (PIAG 1‐05(h)/2007).

## Authors’ contributions

DH, ER and AS planned and implemented the study. ER and AS directed the analysis. SA and TB analysed the data. GC, UM and RP commented on clinical aspects. DH and ER drafted the manuscript, which was approved by all authors.

## Competing interests

GC is in receipt of honoraria and research funding from Janssen, Celgene, Takeda, Sanofi, Bristol Myers Squibb, and is a member of the Speakers Bureau; all remaining authors declare that they have no competing interests.

## Supporting information


**Table SI.** Referral types, GP involvement and definitions.
