# Supplementary material for: Multiple myeloma: routes to diagnosis, clinical characteristics and survival – findings from a UK population‐based study
Source: Br J Haematol. 2017 Feb 1;177(1):67–71. doi: 10.1111/bjh.14513 (PMC5396308; doi:10.1111/bjh.14513)
Supplement: Supplementary file 1 — Table SI. Referral types, GP involvement and definitions. [file BJH-177-67-s001.docx]

**Table SI: Referral types, GP involvement and definitions**

| **Route^1^** | **GP initiated** | **Definition** |
| --- | --- | --- |
| **Emergency presentation** | | |
| Self-referral to A&E | No | Patient’s decision to attend A&E |
| Other to A&E | No | Patient brought to A&E by ambulance crew/police, or nursing home etc. |
| Hospital speciality to A&E | No | Patient referral direct to A&E by other hospital speciality (e.g. outpatient radiology) |
| Unknown referral to A&E | Unknown | Patient presents at A&E but no evidence in medical records of route |
| GP referral to A&E | Yes | GP referral direct to A&E/another acute unit (e.g. acute admissions) |
| GP direct admission to ward | Yes | GP direct admission to hospital ward (i.e. non-A&E) |
| **Non-emergency presentation (NHS, private, out of hours and drop in centre referrals)** | | |
| Two-Week Wait (TWW) | Yes | GP referral to hospital specialist using 2WW criteria – i.e. cancer suspected |
| Urgent referral | Yes | GP referral to hospital specialist stated ‘urgent’ – i.e. cancer not suspected |
| Routine referral | Yes | GP referral to hospital specialist stating ‘routine’ – i.e. cancer not suspected |
| Consultant-to-consultant | No | Hospital consultant to hospital consultant   - As part of work-up towards diagnosis of the haematological malignancy - From a hospital specialist (existing condition) to different specialist (new abnormality) - Includes outpatient appointments and inpatient consultations   Hospital consultant to GP   - requesting onward referral to another speciality   Community specialist to hospital consultant   - Ophthalmologist to hospital consultant |
| **Other** | | |
| Previous diagnosis | N/A | Already under haematology for other condition (suspected malignancy/precursor disease) |
| Route could not be assigned | Unknown | No information in medical records about referral type, including private referrals |

^1^Categories are based on the UK Routes to Diagnosis study (Elliss-Brookes *et al*, 2012), with minor amendments including: separate presentation of the GP urgent and routine referrals; use of consultant-to-consultant route rather than ‘Other outpatient’; omission of ‘Screen detected’, ‘Inpatient elective’ (i.e. no earlier admission found before admission from a waiting list), and ‘Death certificate only’ (DCO) categories.
